# High hemoglobin is associated with increased in-hospital death in patients with chronic obstructive pulmonary disease and chronic kidney disease: a retrospective multicenter population-based study

**DOI:** 10.1186/s12890-019-0933-4

**Published:** 2019-09-18

**Authors:** Libin Xu, Yuanhan Chen, Zhen Xie, Qiang He, Shixin Chen, Wenji Wang, Guohui Liu, Yuanjiang Liao, Chen Lu, Li Hao, Jin Sun, Wei Shi, Xinling Liang, on behalf of China collaborative study on AKI (CCS-AKI)

**Affiliations:** 1 The Second School of Clinical Medicine, Southern Medical University, Guangzhou, 510515 China; 2 Division of Nephrology, Guangdong Provincial People’s Hospital, Guangdong Academy of Medical Sciences, Guangzhou, 510080 China; 3 Department of Nephrology, Inner Mongolia People’s Hospital, Hohhot, 010017 China; 4 Department of Dermatology, Sichuan Academy of Medical Sciences and Sichuan Provincial People’s Hospital, Chengdu, 610072 China; 5 Department of Nephrology, Zhejiang Provincial People’s Hospital (People’s Hospital of Hangzhou Medical College), Hangzhou, 310014 China; 6 Division of Preventive Medicine, School of Public Health, Guangzhou Medical University, Guangzhou, 510515 China; 7 Division of Nephrology, Shanghai Ninth People’s Hospital, School of Medicine, Shanghai Jiaotong University, Shanghai, 200030 China; 8 Department of Nephrology, Dongguan People’s Hospital, Dongguan, 523018 China; 9 Department of Nephrology, Chongqing Ninth People’s Hospital, Chongqing, 400700 China; 10 Department of Nephrology, People’s Hospital of Xinjiang Uygur Autonomous Region, Urumqi, 830001 China; 11 Department of Nephrology, Second Hospital of Anhui Medical University, Hefei, 230601 China; 12 Department of Nephrology, Second Hospital of Jilin University, Changchun, 130022 China

**Keywords:** Hemoglobin abnormality, In-hospital mortality, Polycythemia, Population-based study, China collaborative study on acute kidney injury (CCS-AKI), Chronic obstructive pulmonary disease

## Abstract

**Background:**

Chronic kidney disease (CKD) is a common comorbidity of chronic obstructive pulmonary disease (COPD). Although high hemoglobin (Hb) is detrimental to CKD patients, its relationship with poor outcomes in the COPD population has not been reported. This study aimed to investigate the relationship between high Hb and in-hospital mortality and to explore reference Hb intervals in patients with COPD and CKD.

**Methods:**

This retrospective study was multicenter population-based. A total of 47,209 patients who presented with COPD between January 2012 and December 2016 were included. The average Hb level during hospitalization was used as the Hb level. CKD and advanced CKD were defined as estimated glomerular filtration rates < 60 and < 30 ml/min/1.73 m^2^, respectively. The association between Hb level (measured in 1 g/dL intervals) and in-hospital mortality was analyzed in different multivariable logistic regression models by CKD stratification.

**Results:**

The Hb level was decreased in the CKD subgroup. In the non-CKD group, a higher Hb level was not associated with an increased risk of in-hospital death. However, the Hb level and mortality showed a U-shaped relationship in the CKD group. After adjusting for age and Charlson Comorbidity Index, multivariable regression analysis showed that an Hb level > 17 g/dL was associated with an increased risk of death in the CKD group with an odds ratio (OR) of 2.085 (95% CI, 1.019–4.264). Hb > 14 g/dL was related to an increased risk of death in advanced CKD patients (OR, 4.579 (95% CI, 1.243–16.866)).

**Conclusions:**

High Hb is associated with an increased risk of in-hospital death in COPD patients with CKD, especially among those with advanced CKD. In this group of patients, attention should be paid to those with high Hb levels.

**Electronic supplementary material:**

The online version of this article (10.1186/s12890-019-0933-4) contains supplementary material, which is available to authorized users.

## Background

Chronic obstructive pulmonary disease (COPD) is characterized by chronic airflow limitation, inflammation and lung remodeling. In 2020, COPD is projected to rank fifth worldwide in terms of disease burden and third in terms of mortality [1].

Hemoglobin (Hb) abnormalities, including anemia and polycythemia, are common in the COPD population [2–4]. As hypoxia promotes erythropoiesis, COPD has long been recognized as an important cause of secondary polycythemia. Polycythemia contributes to the development of cor pulmonale and pulmonary hypertension, which are linked to poor prognosis [5]. However, polycythemia prevalence rates reported in recent studies are lower than those in earlier studies and range from 6 to 10% in the COPD population [2, 6, 7], likely due to widespread prescription of long-term oxygen therapy [8].

The association of polycythemia and adverse outcomes is not well understood in patients with COPD. Several previous studies indicated a neutral or protective role of polycythemia [2, 6, 7]. In COPD patients with chronic respiratory failure, polycythemic subjects seemed to have a higher survival rate than normocythemic subjects [9]. Similar results were observed in studies using hematocrit as a polycythemic index. In a small sample cohort study, the hematocrit level was comparable in survivors and nonsurvivors [10]. In another COPD cohort including 2524 patients, the 3-year survival was 24% when the hematocrit was < 35 and 70% when the hematocrit was ≥55% [11].

However, the potential detrimental effects of polycythemia have been implicated in several other chronic conditions, one of which is chronic kidney disease (CKD). Serial randomized controlled trials (RCTs) and meta-analyses have demonstrated the risks of relatively higher Hb concentrations. These increased risks include stroke, hypertension, vascular access thrombosis, cardiovascular events, end-stage renal disease and death, and the cutoff values of high Hb did not exceed those of polycythemia [12–16]. Based on this evidence, the anemia guidelines of the Kidney Disease Outcomes Quality Initiative (KDOQI), which were updated in 2007, recommended an upper target of 12 g/dL for Hb and suggested that Hb levels should not exceed 13 g/dL [17]. Later, the 2012 anemia practice guidelines of the Kidney Disease: Improving Global Outcomes (KDIGO) group recommended that the upper target of Hb should be 11.5 g/dL and that Hb levels should not exceed 13 g/dL [18].

CKD is a common comorbidity in the COPD population [4, 8, 19]. Given the effect of CKD on Hb, does CKD affect the Hb distribution among COPD patients? Furthermore, due to the detrimental role of high Hb in the CKD population, does high Hb pose a more serious threat to COPD patients with CKD than to those without CKD? In this hospital population-based study, we aimed to compare the difference in Hb distribution in non-CKD and CKD patients with COPD and then separately study the relationship between high Hb and in-hospital mortality. The reference Hb intervals in the COPD population were also explored.

## Methods

### Study subjects

Electronic data of COPD patients from the 13 hospitals included in the database of the China Collaborative Study on Acute Kidney Injury (CCS-AKI, a multicenter observational study led by Guangdong Provincial People’s Hospital) were retrospectively analyzed. This dataset includes all hospitalized COPD patients with or without AKI. All patients were admitted to the hospital between January 2012 and December 2016, and the electronic data consisted of demographic information, diagnostic determination, and results of serum creatinine and Hb tests. The creatinine values from the centers were calibrated by the Clinical Laboratory Center of Guangdong General Hospital.

**Fig. 1:**
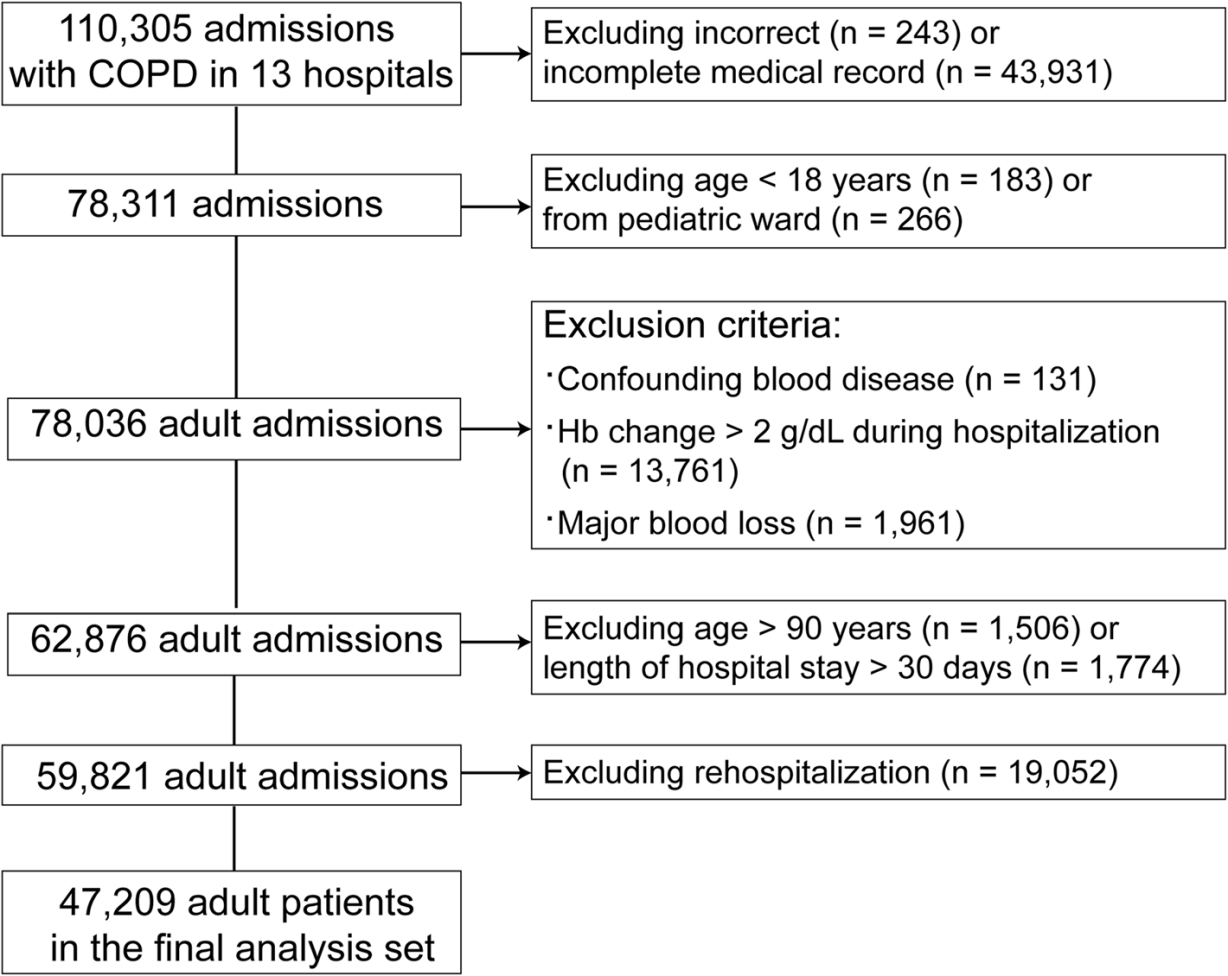
Flow chart of the study population selection

Medical records from 78,036 admissions with COPD suitable for the purpose of this study were screened. COPD and the acute phase were judged according to clinical diagnostic coding. Twelve hospitals were tertiary hospitals, and 1 was a secondary hospital (the detailed information regarding the included hospitals is summarized in Additional file 1: Table S1). The exclusion criteria were as follows: 1) incorrect/incomplete data; 2) < 18 years or from the pediatric ward or > 90 years; 3) anemia that might influence in-hospital death, including myelophthisic anemia, myelodysplastic syndrome, aplastic anemia, thalassemia, hemolytic anemia, or anemia due to significant blood loss; 4) Hb fluctuation (the maximum value minus the minimum value) > 2 g/dL during the hospital stay; and 5) hospital stay > 30 d. For patients with multiple hospitalizations, data from the last admission were used for analysis. The flow chart of population selection is shown in Fig. 1. Ultimately, a total of 47,209 patients were recruited in this study.

### Definitions and outcome

CKD was defined as an estimated glomerular filtration rate (eGFR) < 60 ml/min/1.73 m^2^, which was based on the lowest serum creatinine level within 12 months before discharge of the latest hospitalization, according to the KDOQI guidelines [20]. In-hospital death was the main outcome, and the Hb interval at the minimum death rate was defined as the reference interval. The Charlson Comorbidity Index (CCI) was used as the primary risk index for in-hospital death [2, 21]. Considering the inconsistency in the International Classification of Disease (ICD) standards among the enrolled study centers, ICD codes and diagnostic nomenclature were both used to calculate CCI. The mean Hb level during hospitalization for each patient was used as the Hb level for subsequent analysis.

### Grouping and subgrouping

The enrolled patients were divided into a CKD group and a non-CKD group. To analyze the influence of the severity of CKD on Hb, the CKD group was further divided into an early CKD group (eGFR, 30–59 ml/min/1.73 m^2^) and an advanced CKD group (eGFR < 30 ml/min/1.73 m^2^).

Normally, males have a higher Hb level than females. According to the original design of this study, the patients were also subgrouped based on sex. However, in the CKD population, the male patients did not show a significant difference in Hb levels compared with the female patients. Although females showed slightly higher Hb levels than males during the progression of CKD, no significant difference was observed (Additional file 1: Figure S1). Therefore, stratification based on sex was not taken into consideration during subsequent analysis.

### Statistical analysis

Measurement data are presented as the means ± standard deviation or medians (Q25-Q75). Independent t-tests were used for comparison between groups. Numeration data are presented as percentages (%), and *χ*^2^ tests were used for comparison between groups. Multivariable logistic regression was performed to analyze the risk of in-hospital death, and odds ratios (ORs) and 95% confidential intervals (95% CIs) were calculated. Age and CCI were included as variables. All statistical analyses were performed using SPSS 24.0. A two-tailed *P* value < 0.05 was considered to indicate a significant difference.

## Results

### General data

Of the included 47,209 patients with COPD, 6808 (14.4%) patients also had CKD. Patients in the CKD group were older and had more complications (e.g., cardiovascular diseases and diabetes) than those in the non-CKD group (Table 1).

**Table 1:** Clinical characteristics of the study subjects

	Non-CKD	CKD
Age (years)	68.9 ± 11.9	77.5 ± 8.4
Male [n (%)]	25,624 (63.4%)	4275 (62.8%)
eGFR (ml/min/1.73 m^2^)	88.9 ± 14.9	44.6 ± 13.6
Length of hospital stay (days)	10 (7, 14)	10 (6, 13)
Comorbidity [n (%)]		
Cor pulmonale	12,889 (31.9%)	1860 (27.3%)
Hypertension	13,695 (33.9%)	3621 (53.2%)
Myocardial infarction	1374 (3.4%)	458 (6.7%)
Congestive heart failure	14,883 (36.8%)	3195 (46.9%)
Peripheral disease	6192 (15.3%)	1585 (23.3%)
Cerebrovascular disease	6119 (15.1%)	1545 (22.7%)
Dementia	239 (0.6%)	81 (1.2%)
Connective tissue disease	690 (1.7%)	170 (2.5%)
Peptic ulcer disease	346 (0.9%)	74 (1.1%)
Mild liver disease	4773 (11.8%)	1070 (15.7%)
Diabetes without end-organ damage	4774 (11.8%)	1144 (16.8%)
Hemiplegia	47 (0.1%)	8 (0.1%)
Diabetes with end-organ damage	604 (1.5%)	312 (4.6%)
Tumor without metastasis	3139 (7.8%)	448 (6.6%)
Leukemia	201 (0.5%)	71 (1.0%)
Lymphoma	87 (0.2%)	18 (0.3%)
Moderate or severe liver disease	158 (0.4%)	55 (0.8%)
Metastatic solid tumor	1221 (3.0%)	225 (3.3%)
AIDS	50 (0.1%)	4 (0.1%)
Charlson Comorbidity Index	2 (1, 3)	3 (2, 4)
Medications [n (%)]		
Antibiotics or antifungal agents	21,054 (52.1%)	3705 (54.4%)
ACEIs or ARBs	8034 (19.9%)	1786 (26.2%)
Diuretics^a^	8597 (21.3%)	2123 (31.2%)
Vasoactive drugs	2212 (5.5%)	683 (10.0%)

*eGFR* estimated glomerular filtration rate, *ACEI* angiotensin-converting enzyme inhibitor; *ARB* angiotensin II receptor antagonist

^a^including antihypertensive drug combination

### Hb distribution based on the CKD stratification

**Fig. 2:**
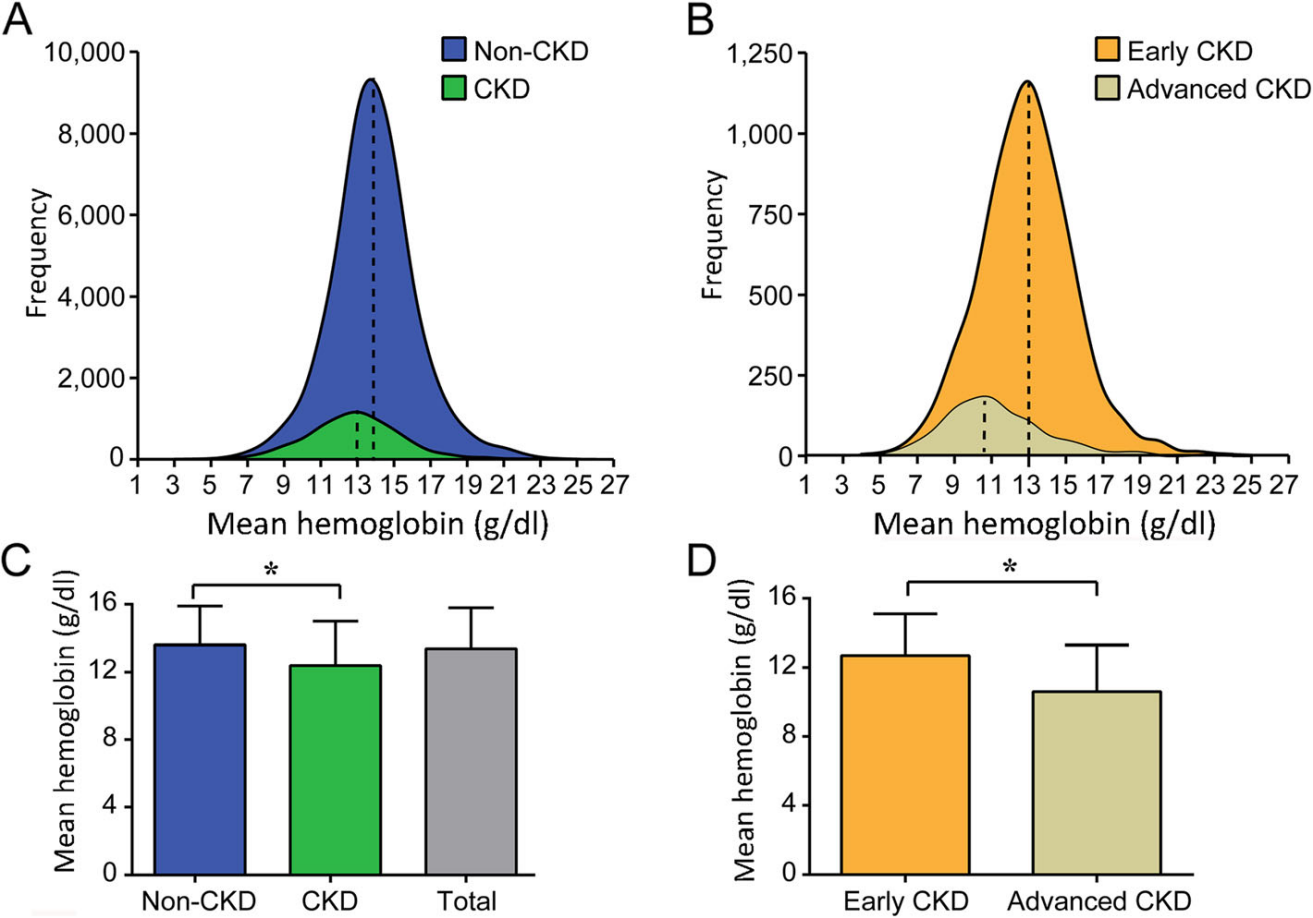
Hb distribution based on CKD stratification. **a**, Compared with the non-CKD group, the CKD group showed a left-shifted Hb distribution curve. **b**, Compared with the early CKD group, the advanced group showed a noticeable left-shifted distribution curve. **c**, The CKD group had lower Hb levels than the non-CKD group. **d**, The advanced CKD group had lower Hb levels than the early CKD group. The dashed lines in panels A and B indicate the median Hb levels of each subgroup. * *P* < 0.001

The average Hb level of the total population was 13.4 ± 2.4 g/dL, with a median (Q25, Q75) of 13.4 (12.0, 14.8) g/dL. Furthermore, Hb distribution frequencies were plotted based on intervals of 1 g/dL according to clinical judgment and preliminary analysis (Fig. 2). The Hb distribution was similar between the non-CKD group and the total population. However, the CKD group showed a left-shifted distribution curve, particularly the advanced CKD group.

### In-hospital death risk based on different Hb intervals

A total of 640 patients (1.4%) died during their hospital stays. The death rates of the non-CKD group and CKD group were 1.0 and 3.3%, respectively, showing a significant difference (χ^2^ = 222.64, *P* < 0.001).

**Fig. 3:**
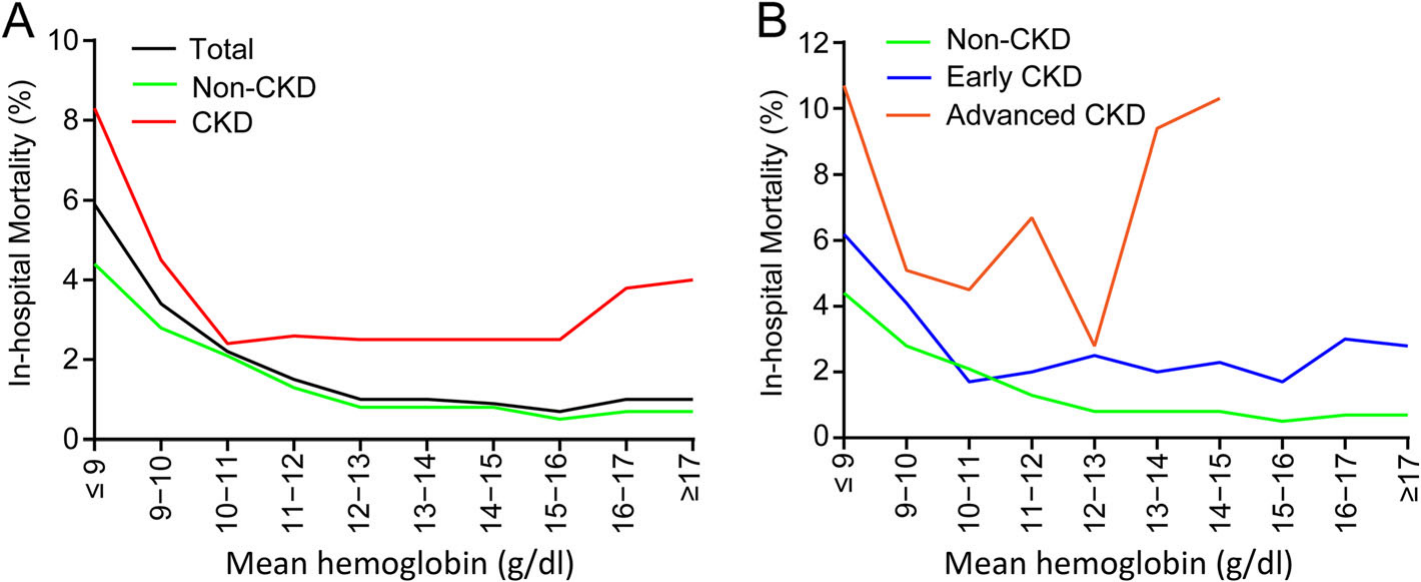
Relation between in-hospital mortality and Hb levels. **a**, The patients were stratified by non-CKD or CKD. **b**, The patients were stratified by non-CKD, early CKD or advanced CKD. As the number of patients with Hb levels > 14 g/dL was small in the advanced CKD group, the groups within intervals above 14 g/dL were merged

Compared with the subgroups with Hb < 10 g/dL, the death rate decreased among those with Hb 10–16 g/dL in both the non-CKD and CKD groups (Fig. 3a). The death rate tended to further decrease in the non-CKD patients with increased Hb. However, in the CKD group, the death rate showed a U-shaped distribution pattern with changes in Hb. Hb levels and mortality rate were inversely proportional when the Hb level was above 16 g/dL (Fig. 3a). Unsurprisingly, this U-shaped pattern was more noticeable in the advanced CKD group (Fig. 3b and Additional file 1: Figure S2).

**Fig. 4:**
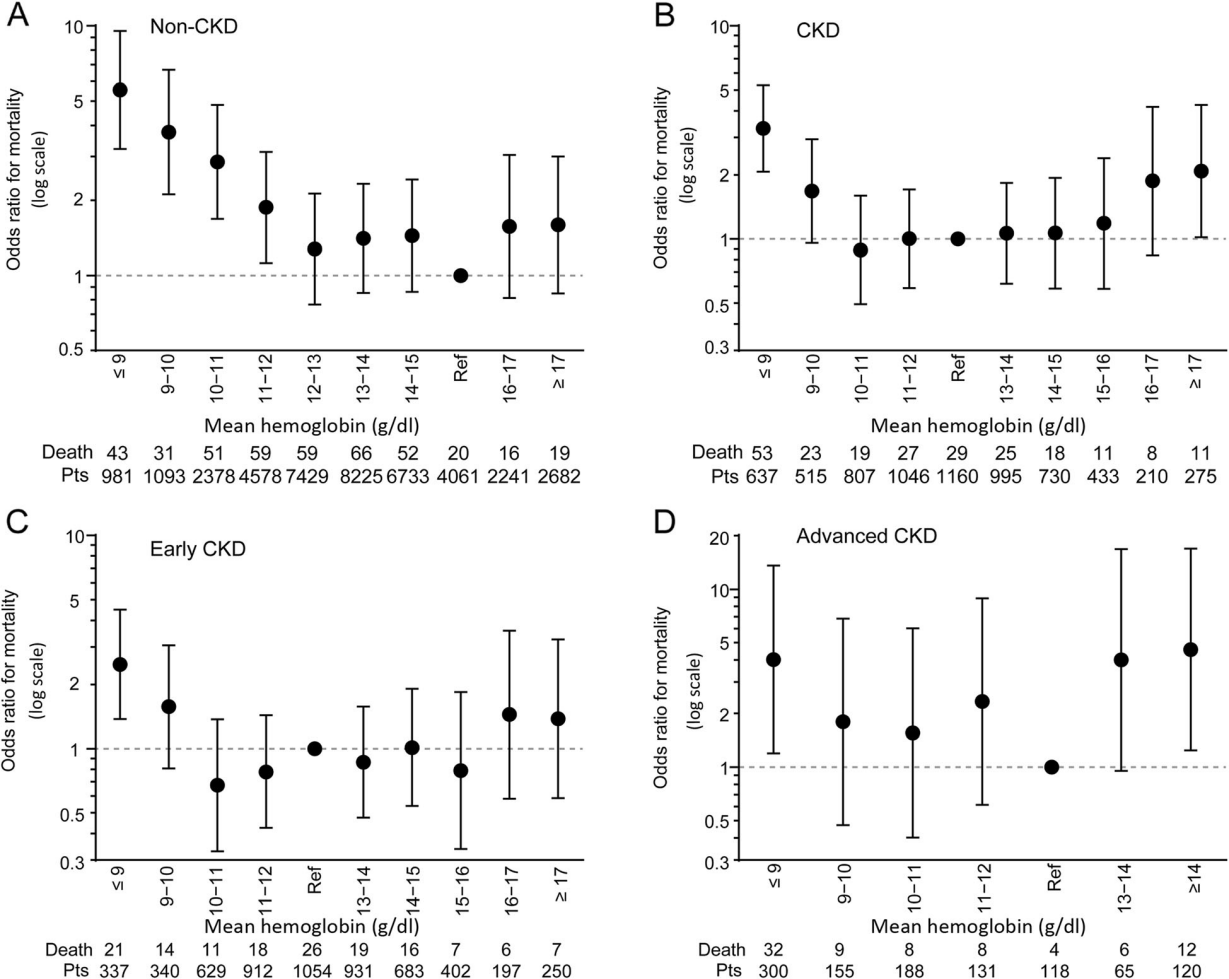
Association between low or high Hb and in-hospital death based on the severity of CKD after multivariable corrections. **a**, Non-CKD. **b**, CKD. **c**, Early CKD. **d**, Advanced CKD (the subgroups with Hb levels > 14 g/dL were merged). The correction variables in the logistic regression model included age stratification (≤55 years, 56–75 years, and > 75 years) and CCI scores (every 1-point increase) (Enter Model). Bars represent the ORs and 95% confidence intervals

Furthermore, the association between a high Hb level and in-hospital mortality was tested using a multivariable logistic regression model that included age (≤55 years, 56–75 years and > 75 years) and CCI scores as the covariates. The outcomes of the multivariable analysis were similar to those of the univariate analysis. For the non-CKD group, although mortality increased when the Hb level was below the 12–13 g/dL interval (the reference interval), a higher Hb level above this interval was not significantly associated with increased mortality (Fig. 4a). For the CKD group, however, Hb levels > 17 g/dL were associated with a higher risk of death, with an OR of 2.085 (95% CI 1.019–4.264, *P* = 0.044) (Fig. 4b). The analysis based on the severity of CKD showed no association between a high Hb level and in-hospital mortality in the early CKD group (Fig. 4c). In the advanced CKD group, however, the mortality rates associated with Hb levels of 15–16 g/dL, 16–17 g/dL and > 17 g/dL were 4.8–8.8 times higher than those associated with Hb levels within the reference interval (Additional file 1: Figure S3). After the subgroups with Hb levels > 14 g/dL were merged, the mortality ORs corresponding to Hb levels of 13–14 g/dL and > 14 g/dL were 4.000 (95% CI, 0.954–16.772, *P* = 0.058) and 4.579 (95% CI, 1.243–16.866, *P* = 0.022), respectively (Fig. 4d).

## Discussion

The results of the present study of the hospitalized COPD population are in accordance with those of other reports showing the effect of CKD on anemia [4] and the associations of anemia with increased mortality [2]. We extended these previous observations by showing the association between high Hb concentration and in-hospital death in CKD patients, which was particularly significant in the advanced CKD group. Based on the analysis of the serial Hb intervals, the upper reference interval of Hb was 17 g/dL for CKD and 14 g/dL for advanced CKD. According to these preliminary reference intervals, high Hb levels were observed in 275 patients (4.0%) with CKD and 116 patients (10.8%) with advanced CKD.

A high Hb concentration is double-edged. It not only is considered an adaptive physiological response to hypoxemia in the COPD population [22] but also plays a detrimental role in CKD [12–16]. The mechanism of the association between high Hb and death is unclear. Several hypotheses have been suggested, including increased blood volume and viscosity and improved platelet function after correction of anemia [23, 24]. These mechanisms cause hyperviscosity of the blood and contribute to increased pulmonary vascular resistance. Thromboembolic disease attributed to polycythemia affecting the pulmonary vascular bed again increases pulmonary vascular resistance. Polycythemia also inhibits the endothelial-dependent relaxation response to acetylcholine [25, 26]. In addition, erythropoiesis-stimulating agents and iron may play detrimental roles independent of their therapeutic roles in hemopoiesis [14, 24, 27–30].

No previous studies have reported a clinical threat of a high Hb level in COPD patients [6, 7, 31]. In such studies, the authors defined a high Hb level based on the standard for polycythemia, i.e., > 17 g/dL for males and > 15 g/dL for females. The study subjects were primarily stable COPD outpatients whose pathogenetic conditions were less severe and who had a lower mortality than inpatients, and patients with heart disease, kidney disease and malignancies were often excluded, as these conditions might serve as confounding diseases of COPD [6, 7, 31]. Compared with these studies, the current study showed the following noticeable differences: 1) The current study adopted Hb intervals of 1 g/dL, rather than the polycythemia standard, to explore a possible reference Hb level based on the mortality of patients within different Hb intervals. 2) The current study did not exclude COPD patients with other diseases except for hematologic disease and hemorrhagic disease, which could possibly have had an influence on mortality, making the study subjects more representative. 3) The CCI was used for correction of the influence of multiple complications on mortality, although the small number of death-related residual confounding factors of the COPD patients was not corrected for [8]; the scoring system successfully covered most of the death-related comorbidities. 4) Most importantly, this study adopted the presence of CKD and the severity of CKD as stratification standards. These features not only explain the major differences between this study and previous studies but also allow the current team to move forward to explore the upper reference limit for Hb in COPD patients.

The exploration of the optimal upper Hb target in the CKD population has gone through a long journey. In early studies, exploring the optimal Hb target was difficult due to the considerable heterogeneity in the criterion for high Hb [16]. Only in recent years have a number of representative registered clinical trials (the Cardiovascular Risk Reduction by Early Anemia Treatment with Epoetin Beta (CREATE), the Correction of Hemoglobin and Outcomes in Renal Insufficiency (CHOIR) and the Trial to Reduce Cardiovascular Events with Aranesp Therapy (TREAT)) reported higher target Hb concentrations of 13–15, 13.5 and 13.5 g/dL, respectively [12–14]. The results of these studies demonstrated the association between a higher Hb target level and a higher death risk and became the important basis for the KDOQI and KDIGO guidelines. A meta-analysis that summarized 9 registered clinical trials, where the high Hb targets were set between 13 g/dL and 16 g/dL, showed that an Hb target within 12–16 g/dL was associated with a high risk of death [15]. However, none of these trials focused on the COPD population. In this study, we investigated the risks associated with high Hb in a COPD population based on CKD stratification for the first time. While exploring the upper reference limit for Hb, the current team adopted a stratification method to avoid the interaction of CKD with abnormal Hb levels. The results of this study showed that the guidelines regarding the upper reference limit for Hb in the COPD population with CKD were similar to those in the CKD population, suggesting that management of anemia for CKD patients may serve as a reference for the treatment of COPD complicated with CKD and that the upper reference limit for Hb should also be taken into consideration. These results provide evidence for future work on optimal Hb limits.

This study has the following limitations. First, the CCS-AKI databank suffers from a lack of information regarding erythropoietin- and COPD-related medicine and hematologic testing indices except for Hb, as the initial designed endpoint of the databank was renal events. Therefore, the potential influence of these factors on mortality failed to be corrected for. Second, because the data were derived from retrospective electronic medical records, supplemental oxygen use, pulmonary function and blood gas analysis indices were not available, and the severity of hypoxia and patient conditions were not evaluated. Thus, the impact of a high Hb level could not be assessed independent of the severity of COPD. In addition, approximately 40% of subjects were excluded due to incorrect or incomplete medical records (Fig. 1), which might cause selection bias. Third, the adverse outcome observed in this study was limited to in-hospital mortality, and other adverse events, such as quality of life, cardiovascular events, shock and long-term death, were not included. Therefore, the possibility that the mechanisms underlying the influence of high Hb on these adverse events were different from those underlying mortality cannot be excluded. Finally, Hb levels in actual clinical practice may not be consistent with the target limits for Hb management. Therefore, the optimal Hb target limit for the COPD population with CKD remains to be confirmed in future studies.

## Conclusions

In conclusion, high Hb was associated with an increased risk of in-hospital death in the COPD population with CKD, especially those COPD patients with advanced CKD. The management of erythrocyte abnormalities in COPD should not be limited to anemia, but due attention should be given to CKD stratification and increased Hb levels.

## Additional file

Additional file 1: **Table S1.** Information on the enrolled hospitals. **Figure S1.** Comparison of the Hb levels between males and females in the COPD population complicated with CKD. **Figure S2.** In-hospital mortality at different Hb levels using uncombined data in the advanced CKD group. The subgroups of the advanced CKD group with Hb levels > 14 g/dL were not merged. In the advanced CKD group, the mortalities of the subgroups with Hb levels of 13–14 g/dL, 14–15 g/dL, 15–16 g/dL, 16–17 g/dL and higher than 17 g/dL were 9.4, 4.3, 12.9, 15.4, and 16.0%, respectively. Points a, b and c indicate the lowest mortalities of the non-CKD group (0.5%), the early CKD group (1.7%), and the advanced CKD group (2.8%), respectively. **Figure S3.** Association between low or high Hb levels and in-hospital death using uncombined data in the advanced CKD group. The subgroups with Hb levels > 14 g/dL were not merged. Compared with the reference interval (12–13 g/dL), Hb levels of 15–16 g/dL, 16–17 g/dL and > 17 g/dL significantly increased patient mortality, with ORs of 5.831 (95% CI, 1.209–28.113), 7.417 (95% CI, 1.083–50.796) and 8.781 (95% CI, 1.781–43.284), respectively. Bars represent the ORs and 95% confidence intervals. (DOC 1185 kb)

## Data Availability

All data generated or analyzed during this study are included in this article and its supplementary information files.

## Abbreviations

CKD: Chronic kidney disease
COPD: Chronic obstructive pulmonary disease
Hb: Hemoglobin
KDOQI: Kidney Disease Outcomes Quality Initiative
