# Supplementary material for: High hemoglobin is associated with increased in-hospital death in patients with chronic obstructive pulmonary disease and chronic kidney disease: a retrospective multicenter population-based study
Source: BMC Pulm Med. 2019 Sep 18;19:174. doi: 10.1186/s12890-019-0933-4 (PMC6749661; doi:10.1186/s12890-019-0933-4)
Supplement: Supplementary file 1 — Table S1. Information on the enrolled hospitals. Figure S1. Comparison of the Hb levels between males and females in the COPD population complicated with CKD. Figure S2. In-hospital mortality at different Hb levels using uncombined data in the advanced CKD group. The subgroups of the advanced CKD group with Hb levels > 14 g/dL were not merged. In the advanced CKD group, the mortalities of the subgroups with Hb levels of 13–14 g/dL, 14–15 g/dL, 15–16 g/dL, 16–17 g/dL and higher than 17 g/dL were 9.4, 4.3, 12.9, 15.4, and 16.0%, respectively. Points a, b and c indicate the lowest mortalities of the non-CKD group (0.5%), the early CKD group (1.7%), and the advanced CKD group (2.8%), respectively. Figure S3. Association between low or high Hb levels and in-hospital death using uncombined data in the advanced CKD group. The subgroups with Hb levels > 14 g/dL were not merged. Compared with the reference interval (12–13 g/dL), Hb levels of 15–16 g/dL, 16–17 g/dL and > 17 g/dL significantly increased patient mortality, with ORs of 5.831 (95% CI, 1.209–28.113), 7.417 (95% CI, 1.083–50.796) and 8.781 (95% CI, 1.781–43.284), respectively. Bars represent the ORs and 95% confidence intervals. (DOC 1185 kb) [file 12890_2019_933_MOESM1_ESM.doc]

**Table S1.** Information on the enrolled hospitals

| Hospital | Hospital Level | Province | Enrolled Patients Number | % |
| --- | --- | --- | --- | --- |
| Guangdong General Hospital | Tertiary | Guangdong | 2,817 | 6.0 |
| Kashi First Hospital | Tertiary | Xinjiang | 8,676 | 18.4 |
| Lufeng People’s Hospital | Secondary | Guangdong | 304 | 0.6 |
| Inner Mongolia People's Hospital | Tertiary | Inner Mongolia | 7,138 | 15.1 |
| Chongqing Ninth People's Hospital | Tertiary | Chongqing | 5,951 | 12.6 |
| Sichuan Provincial People’s Hospital | Tertiary | Sichuan | 6,271 | 13.3 |
| Zhejiang Provincial People’s Hospital | Tertiary | Zhejiang | 367 | 0.8 |
| Shanghai Ninth People's Hospital | Tertiary | Shanghai | 587 | 1.2 |
| The Second Hospital of Anhui Medical University | Tertiary | Anhui | 3,116 | 6.6 |
| People’s Hospital of Xinjiang Uygur Autonomous Region | Tertiary | Xinjiang | 1,170 | 2.5 |
| Hohhot First Hospital | Tertiary | Inner Mongolia | 5,254 | 11.1 |
| The Second Hospital of Jilin University | Tertiary | Jilin | 2,191 | 4.6 |
| Dongguan People’s Hospital | Tertiary | Guangdong | 3,367 | 7.1 |
| Total |  |  | 47,209 | 100.0 |


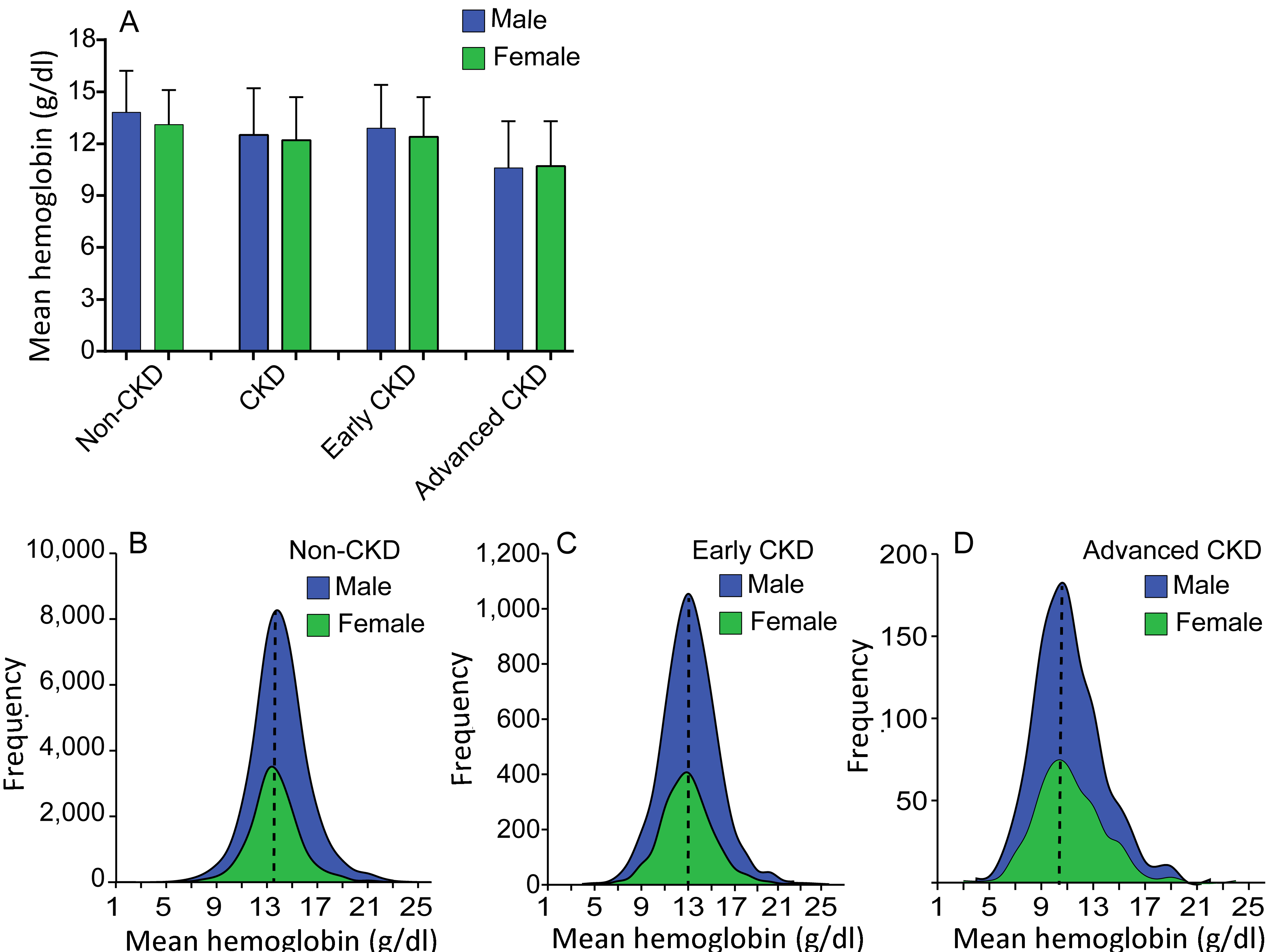


Figure S1. Comparison of the Hb levels between males and females in the COPD population complicated with CKD.


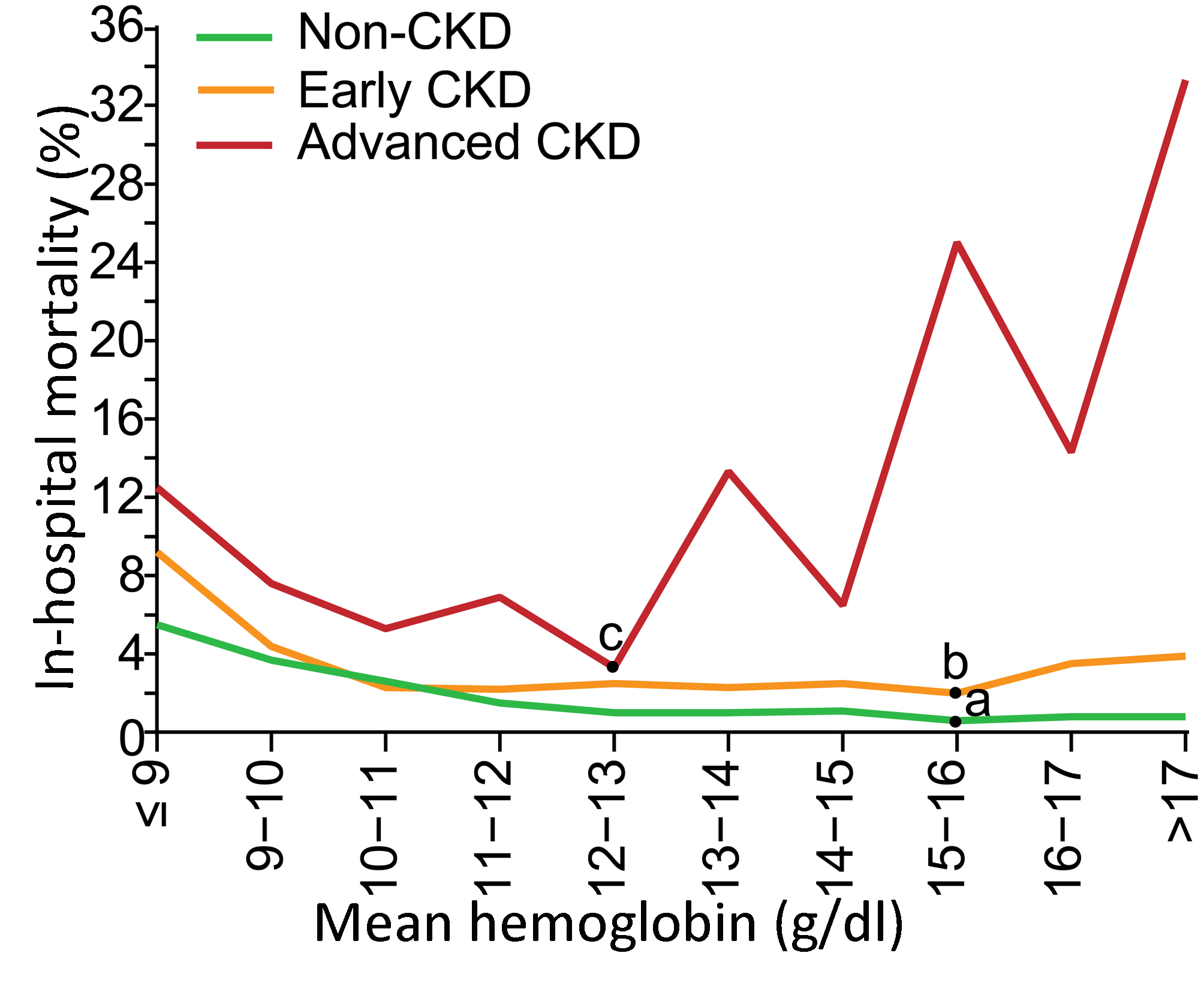


Figure S2. In-hospital mortality at different Hb levels using uncombined data in the advanced CKD group. The subgroups of the advanced CKD group with Hb levels > 14 g/dL were not merged. In the advanced CKD group, the mortalities of the subgroups with Hb levels of 13-14 g/dL, 14-15 g/dL, 15-16 g/dL, 16-17 g/dL and higher than 17 g/dL were 9.4%, 4.3%, 12.9%, 15.4%, and 16.0%, respectively. Points a, b and c indicate the lowest mortalities of the non-CKD group (0.5%), the early CKD group (1.7%), and the advanced CKD group (2.8%), respectively.

**
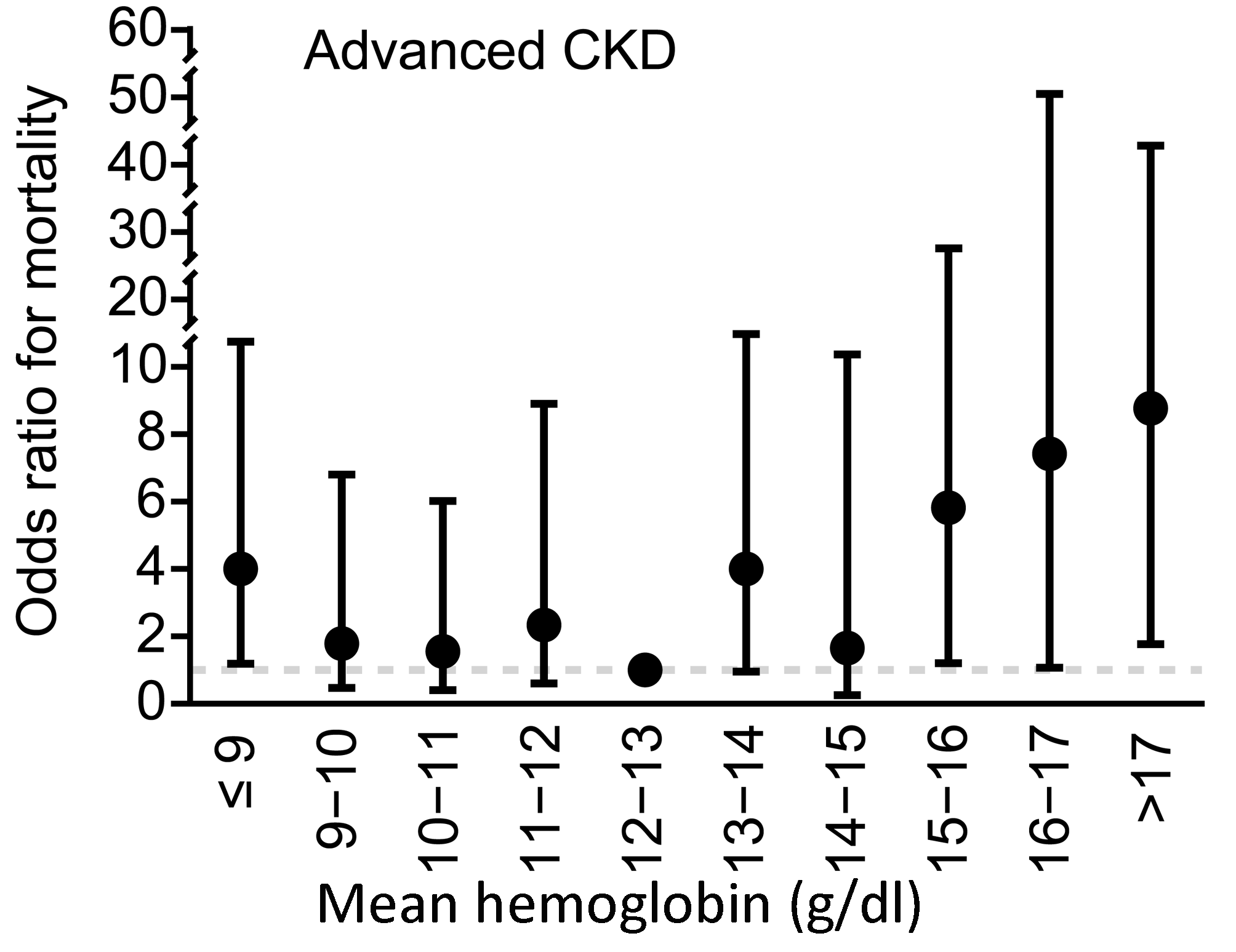
**

Figure S3. Association between low or high Hb levels and in-hospital death using uncombined data in the advanced CKD group. The subgroups with Hb levels >14 g/dL were not merged. Compared with the reference interval (12-13 g/dL), Hb levels of 15-16 g/dL, 16-17 g/dL and > 17 g/dL significantly increased patient mortality, with ORs of 5.831 (95% CI, 1.209-28.113), 7.417 (95% CI, 1.083-50.796) and 8.781 (95% CI, 1.781-43.284), respectively. Bars represent the ORs and 95% confidence intervals.
